# Unreduced Dislocations of the Femur

**Published:** 1890-07-19

**Authors:** 


### SURGERY.

### UNREDUCED DISLOCATIONS OF THE FEMUR.

No advance in modern surgery is more thorough than that made in the treatment of dislocations. The change in treatment founded on an accurate knowledge of the causation of these injuries and the nature and exact description of the lesions of the soft parts accompanying them, is the result of no striking operative method, but of patient clinical and pathological observation. In this field, as in so many others, the name of Astley Cooper rises as a landmark, and no stronger evidence of the superiority of the surgery of this generation over that preceding his could be adduced than is offered by the fact that Cooper was able to supply the museum now existing at St. Thomas's Hospital with specimens of old unreduced dislocations on to the dorsum ilii, into the obturator foramen and on to the pubes in the course of a few years. When we pride ourselves on the change which has taken place, however, we must bear in mind that, beyond the heritage we have received from our predecessors, derived from careful observation both of patients and post-mortem specimens, we possess a no less remarkable advantage in our power of putting muscular resistance out of the question as an obstacle to reduction by the use of anæsthetics. These not only allow of a more satisfactory examination of the joint, but have practically abolished the occurrence of many injuries often inflicted on patients by the efforts of the surgeon at reduction by forcible extension methods. By the aid of these various improvements, it has been happily of late years a rare event indeed to meet with an unreduced dislocation, unless the patient has been unfortunately out of reach of proper immediate attendance. Nevertheless, such cases are still met with occasionally, and in the treatment of these, the confidence now justly entertained of our power to perform large operations without inducing suppuration has opened a field to the modern surgeon in arthrotomy, or, excision of the affected joint. Professor Oscar Bloch, of Copenhagen, has recently contributed to the *Revue D'Orthopédie** an original memoir on the subject of old unreduced dislocations of the hip joint, with an analysis of the results of operations performed for their relief. Professor Bloch has collected 14 cases from various sources, to which is added a recent one of his own.

Of these, in four cases tenotomy was the procedure chosen, in two subcutaneous, without result (Hamilton. MacCormac), in two in an open wound (Vecelli), both successful.

In one (Polaillon), arthrotomy was performed at the end of six weeks, with success as far as reposition was concerned, but the patient died from acute traumatic gangrene.

In two, subtrochanteric osteotomy was performed, one giving a good result, and one failing to better the patient (Wahl. Koch).

Excision was performed in eight cases with uniform success; in one case the head was necrosed as a result of fracture.

Of the fifteen dislocations nine were dorsal, four obturator, and two sciatic.

The indications for operation were in one case only neuralgia, that of Delagarde, performed in 1866, and recorded in St. Bartholomew's Hospital reports. In all the others the operation was decided on in consequence of defective progression.

Subcutaneous tenotomy has proved the most unsuccessful measure, failing in every case, and this is scarcely to be wondered at when the condition of the acetabular cavity discovered in the excisions is remembered. But in more recent cases tenotomy would scarcely seem to hold out any very great chance of reduction, and the plan adopted by Hamilton of dividing the ilio-femoral ligament by removing the fulcrum employed in the ordinary manipulative manœuvres would prevent any chance of reduction except by the extension method. Two cases in which tenotomy was combined with an open wound (Vecelli) gave good results, but here, unfortunately, details are wanting. The importance of these cases, however, principally depends on the support they give to arthrotomy, which, as Professor Bloch justly remarks, is the proper antecedent to every excision. Arthrotomy, however, as illustrated by the results published, is unpromising; in only one case, and that one of six weeks' standing, was reposition of the head effected, although it was the preliminary procedure in most of the cases of excision. Subtrochanteric osteotomy was tried in two cases only, and in one failed. Professor Bloch is apparently no advocate for this mode of treatment, but it may be remarked that theoretically it offers advantages that may be yet practically proved. These lie in the fact that a moveable excised hip is not a common result, while we know that the displaced head of the femur is capable of setting up such local changes as may lead to the development of a good acetabular cavity. A subtrochanteric osteotomy, therefore, besides being a far less severe measure than excision, would give not only a result ensuring a wider basis of support to the pelvis, but also a chance of very fair movement of the limb.

* Remarques a propos d'un cas de luxation irréductible de la hanche, traité par opération. Tome 1. No. 3.

Professor Bloch adds some interesting remarks on the causes of irreducibility of certain luxations. He points out that the length of existence is not the chief factor, since dislocations have been reduced at very variable periods, and some beyond the extreme limit of eight weeks fixed by Cooper, and still very generally accepted, while others of quite recent date have proved intractable to experienced surgeons. Interposition of a part of the capsule, smallness of rent in the capsule, and fracture of the neck of the femur are discussed, and the materials offered by the fifteen cases reviewed. This review brings out in strong light the fact that in six of the cases the cotyloid cavity was practically non-existent, being filled up with fibrous tissue, or granulation material. The acetabulum having become of no further use in fact, organisation of blood clot had rapidly obliterated it. In the cases of Volkmann and MacCormac, the development of firm scar tissue about the articulation proved a further obstacle to replacement of the femur, even with the joint fully opened.

Lastly, the date at which operative measures in any given case may be taken into consideration is discussed. Professor Bloch rightly defers the raising of the question to one or one and a half to two months after the accident, and even if an arthrotomy is attempted it certainly should not be performed before that date, for, as he points out, in many cases a joint has been restored to its natural position after repeated trials by the same surgeon, and, except in the case of a concurrent fracture of the neck of the femur, we are always ignorant of the precise cause of the irreducibility.

Pain and incapacity for work are the most urgent indications. It must be remarked, however, as to the latter that for a patient who can wait, in many cases time for adaptation to new conditions and the formation of a false joint may give almost as good a result as excision. Hence no inflexible rule can be laid down on the matter. Professor Bloch would limit the employment of subtrochanteric osteotomy to cases in which change in the axis of the limb is the chief factor, but this would seem to cover most of the cases coming into the second category. He remarks, however, that the troubles liable to follow the necessary fixation of the pelvis and the luxation are unfavourable moments in the carrying out of this treatment. Should excision be chosen pure decapitation is rightly recommended, and the apposition of the neck to the acetabulum, in which in his own case he made a preparatory depression by removing some of the tissue filling the cavity, with success.

